# Scaling a Hydraulic Motor for Minimally Invasive Medical Devices

**DOI:** 10.3390/mi15010131

**Published:** 2024-01-12

**Authors:** Manjeera Vinnakota, Kishan Bellur, Sandra L. Starnes, Mark J. Schulz

**Affiliations:** 1 College of Engineering and Applied Sciences, University of Cincinnati, Cincinnati, OH 45221, USA; vinnakbm@mail.uc.edu (M.V.); schulzmk@ucmail.uc.edu (M.J.S.); 2 College of Medicine, University of Cincinnati, Cincinnati, OH 45221, USA; starnesl@ucmail.uc.edu

**Keywords:** miniature hydraulic motor, 3D printing, minimally invasive surgery, numerical modeling, ANSYS CFX, fluid structure interaction

## Abstract

Aligned with the medical device industry’s trend of miniaturization, academic and commercial researchers are constantly attempting to reduce device sizes. Many applications require miniature actuators (2 mm range) to perform mechanical work; however, biocompatible micromotors are not readily available. To that end, a hydraulic motor-driven cutting module that aims to combine cutting and drug delivery is presented. The hydraulic motor prototype developed has an outside diameter (OD) of ~4 mm (twice the target size) and a 1 mm drive shaft to attach a cutter. Four different designs were explored and fabricated using additive manufacturing. The benchtop experimental data of the prototypes are presented herein. For the prototype motor with fluid inlet perpendicular to the blades, the average angular velocity was 10,593 RPM at a flowrate of 3.6 mL/s and 42,597 RPM at 10.1 mL/s. This design was numerically modeled using 3D-transient simulations in ANSYS CFX (version 2022 R2) to determine the performance characteristics and the internal resistance of the motor. Simplified mathematical models were also used to compute and compare the peak torque with the simulation estimates. The viability of current design represents a crucial milestone in scaling the hydraulic motor to a 2 mm OD to power a microcutter.

## 1. Introduction

Miniaturization is a trend in the medical device industry with the goal of reducing device sizes while maintaining function. As more procedures become less or non-invasive, patients benefit from shorter hospital stays and less pain. Due to the size restrictions of certain medical applications, a wide variety of minimally invasive instruments with a smaller than 5 mm outside diameter have been developed [1,2,3]. In general, pediatric and neonatal applications need smaller instruments [4,5,6]. Some applications in adults also have severe size constraints because of anatomical limitations. For example, peripheral airways in the lungs are 2 mm or less in diameter [7], ureters are 2 to 4 mm in diameter [8,9], the common biliary duct has an average diameter of ~5 mm [10,11,12], and pancreatic ducts are in the 3 mm size range [13]. Therefore, any device designed to navigate these narrow lumina for diagnosis or therapy should be small, flexible, and conforming to the anatomical boundaries. Another sizing consideration comes from compatibility with existing standardized instrumentation such as endoscopes, bronchoscopes, needles, etc. New minimally invasive devices being developed must comply with the industry sizing standards. For instance, the diagnostic cardiac catheters typically come in 3 to 8 French sizing, which are 1 to 3 mm in diameter [14,15,16]. For utilizing or retrofitting the tendon based steering mechanism of a commercial steerable catheter, any prototype end effector under development should match the catheter sizing.

The ability to perform mechanical work becomes more challenging as the device size reduces. Further, the reach of the device is limited by its dimensions. Untethered microrobots and microdevices provide a lucrative solution to the problem of reach but are at a very nascent stage for in vivo trials [17,18,19]. A combination platform where an existing system (robotic or tethered device) is used for reaching close to the target and then deploying the untethered microdevices was proposed in the literature [20,21,22,23]. This approach relies on the ability of the tethered device to be flexible and have macro tissue manipulation abilities that microdevices lack.

For applications with size constraints, such as those mentioned above, an actuator smaller than 4 mm is desirable. Several motors/actuators with a variety of working principles such as electric, hydraulic, ultrasonic, electro conjugate fluids, etc., have been designed to cater to this need. Electric motors 4 mm in diameter and smaller are commercially available from multiple manufacturers like Maxon International Ltd., Sachseln, Switzerland (multiple model numbers [24]), Pelonis Technologies Inc., Exton, PA, USA (Model PT0408-111-Q [25]), and Faulhaber, Schönaich, Germany (Model 0308H003B [26]). The smallest electric motor is a 0.6 mm diameter brushless motor from Orbray, Co., Ltd., Tokyo, Japan [27]. However, there are unknowns regarding biocompatibility and how the waste heat interacts with tissue.

Product offering from Interscope Inc. (Northbridge, MA, USA) is of particular interest as it utilizes a small hydraulic turbine and is designed to work inside an endoscope channel with a device outside diameter 4 to 5 mm [28]. There is no heat involved in tissue cutting due to the device’s hydraulic operation. The prototype miniature hydraulic motor presented in this paper is also ~4 mm OD and will be further scaled down to ~2 mm OD. Another option for mechanical work output is an ultrasonic micromotor. The smallest ultrasonic micromotor developed by Furukawa, Y., et al. [29] has an outside diameter of 0.95 mm. One of the motor configurations has a hollow shaft that can potentially be used for drug delivery. The wear of the components is a concern as the stator and rotor are in contact during rotation. Watson, B., et al. [30] developed an ultrasonic micromotor for in vivo application with an overall diameter of 400 µm. In the review paper by Mashimo, T. [31], the scaling of ultrasonic micromotors was discussed and successfully implemented applications of ultrasonic motors with 4.5 mm OD were presented [32]. Kikuchi, K., et al. [33] claim the smallest stator ultrasonic micromotor, with a 150 µm rotor. Micromotors using electro conjugate fluids (ECF) can also be used for driving micro actuators. These motors were developed by Yokota, S., et al. [34] and a micromotor with ID 5 mm, in the size range of current prototype, was presented [35]. The smallest ECF micromotor by Yokota, S., et al. has a 2.8 mm outside diameter [36].

Though the above list is not exhaustive, it serves as a current state-of-the-art summary of miniature motor options available and those comparable in size to the prototype mini hydraulic motor presented in this paper. Many of the existing motor options, however, require electrical connections and are susceptible to ohmic heating. Biocompatibility for direct tissue contact is also unknown.

We aim to develop a biocompatible, hydraulic micromotor-driven cutting module with drug delivery function with an outside diameter of 2 mm—more than 50% smaller than the prototype presented here. This micromotor is being designed to power a microcutter and has fluid exits for drug or nano particle delivery at the target location. The design presented in this paper is a 2:1 scale (~4 mm OD) of the target size. This serves as a verification of the intermediate design step to show consistent mechanical work output. The learnings will be leveraged to scale down the design further by halving the overall diameter to 2 mm. The paper presents detailed design of the scaled-up motor, design alternatives, fabrication of miniature motor prototypes using additive manufacturing and the experimental setup for testing. The scope of this paper is limited to capturing the design evolution, experimentation, and modeling of scaled motor designs. The mechanical/application testing of 2 mm OD will be detailed in a future publication.

ANSYS CFX is often used by researchers for simulating complex 3-Dimensional transient flows in turbomachinery. Yoon, Y., et al. modeled gear pumps [37], Nishi, Y., et al. modeled the performance of dental air turbines [38] and Juraeva, M., et al. studied the optimization of an air driven dental turbine using a combination of CFX and DOE (design of experiments) [39,40]. Utilizing the fluid structure interaction modeling capabilities available in Ansys CFX, a numerical model was developed for predicting performance characteristics of the 4 mm OD prototype design. The model was validated using the experimental results and a mathematical model. The results are presented in subsequent sections of this paper.

## 2. Materials and Methods

**Figure 1:**
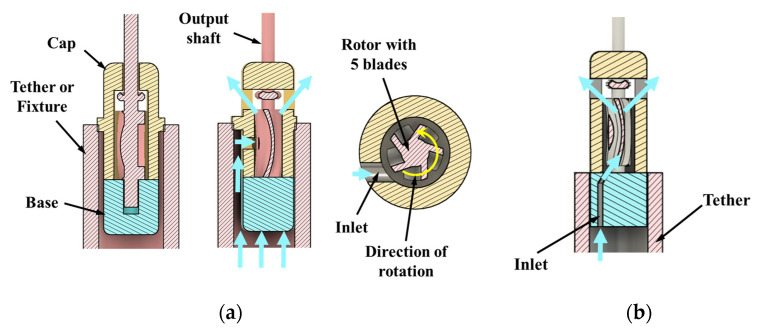
Inlet configurations: (**a**) perpendicular to blades (design option 1); (**b**) tangential inlet (design option 2). Fluid flow lines are shown with blue arrows.

A miniature hydraulic motor based on the principles of an impulse turbine was designed with an outside diameter of 4 mm at the shaft end of the motor. This design is 2:1 scale of the target device outside diameter. The motor consisted of 3 components: cap, base, and rotor (Figure 1). The design had an output shaft of 1 mm to which a cutter can be attached. The rotor blades were 500 µm wide and 5 mm in length. The output shaft was integrated with the rotor blades and a seal. The motor prototype can be operated by both water and air, and these working fluids are biocompatible and readily available in operating rooms.

For miniature devices, simplicity is a key design attribute as the assembly of multiple components becomes very challenging when the component size approaches sub-millimeter length scales. Therefore, multifunctional designs with a minimal number of components are preferred. In case of the hydraulic motor developed, the cutting and drug delivery functions were combined in the same design. The working fluid exiting the device serves as a drug delivery mechanism. Additive manufacturing enabled the production of small complex geometries in the designed components.

**Figure 2:**
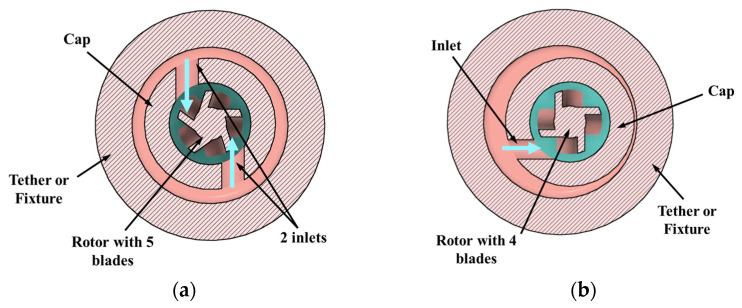
Inlet and rotor configurations: (**a**) two inlets with a 5-blade rotor (design option 3); (**b**) single inlet with a 4-blade rotor (design option 4). The fluid inlet is shown with blue arrows.

Based on the knowledge of impulse turbines, design features such as number of blades, inlet orientation relative to the blade, and number of inlets were identified as critical to the motor performance. These key features were varied to obtain 4 different design options (Figure 1 and Figure 2) that were conducive to prototyping. The four configurations of the miniature motor listed below were prototyped and tested to establish feasibility and baseline performance. The goal was to down select a design and understand features/parameters that would be scaled to the target size of 2 mm OD.

Design option 1: Single inlet design with a 5-blade rotor. In this design the inlet is perpendicular to the blades. This is the baseline design.

Design option 2: Single tangential inlet with a 5-blade rotor. This inlet orientation helps in reducing the overall device diameter.

Design option 3: Dual inlets with a 5-blade rotor. In this design the inlets are perpendicular to the blades. In theory, this design would give higher torque as the energy from two water jets is being transferred to the rotor.

Design option 4: Single inlet design with a 4-blade rotor. In this design, the spatial interaction between jet and blade is different compared to a rotor with 5 blades due to the difference in angular position of the blades. The rotor in the baseline design was replaced with a 4-blade rotor, no other changes were made to the rest of the components.

The CAD cross-sections of baseline design with inlet perpendicular to the blades (option 1) are shown in Figure 1a, and the tangential inlet prototypes (option 2) are shown in Figure 1b. Design options 3 and 4 are shown in Figure 2a,b. Inlet diameter was 850 µm in design options 1, 3, and 4, and 400 µm in option 2. Nominal clearance between rotor and cap was 250 µm in all the designs.

### 2.1. Prototypes

**Figure 3:**
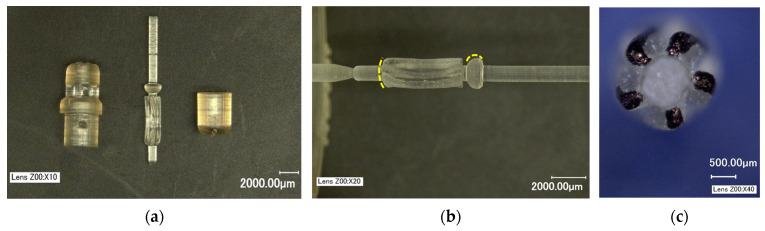
The 4 mm OD prototype components: (**a**) 3D-printed parts using biocompatible materials; (**b**) rotor printed with a single support and the yellow lines highlight the rounded features due to the surface tension of liquid resin. (**c**) bottom view of the rotor with blades colored black for contrast.

Formlabs Form 3+ printers (Formlabs, Somerville, MA, USA) were used for 3D printing the prototype components in two different materials: (1) standard white (LGPWH04) and (2) biocompatible resin (FLBMAM01). Prototype parts are shown in Figure 3a. The number of supports were minimized to avoid damage during post-processing, and the rotor was printed using only one support (Figure 3b). The build direction was perpendicular to the component axis. As the features get smaller, the surface tension effects of liquid resin play a crucial role in obtaining crisp feature definition. The rounding of sharp edges and convexity of the bottom surface were visible in the rotor (Figure 3b). Figure 3c shows the bottom view of the 5 blades which were colored black for contrast.

The XY resolution for the Form3+ printer is 25 µm [41]. Since the components were tall in Z direction, as the layers build up in Z, there was a stack-up of tolerance resulting in form errors such as perpendicularity of axis w.r.t blades. This resulted in the eccentric rotation of shaft during operation. For the current study, this was acceptable, but it is worth mentioning that feature accuracy can be improved by micro-additive manufacturing. A BMF microArch S-140 printer (BMF, Maynard, MA, USA) with a XY positional accuracy of 1 µm [42] was successfully used to prototype preliminary to-scale 2 mm OD hydraulic micromotor designs and will be used for final device prototyping in the future. Another way to increase the component accuracy is to utilize supportless printing, thereby eliminating the introduction of imperfections during post-processing. The results of the 2 mm prototype are beyond the scope of this paper.

**Figure 4:**
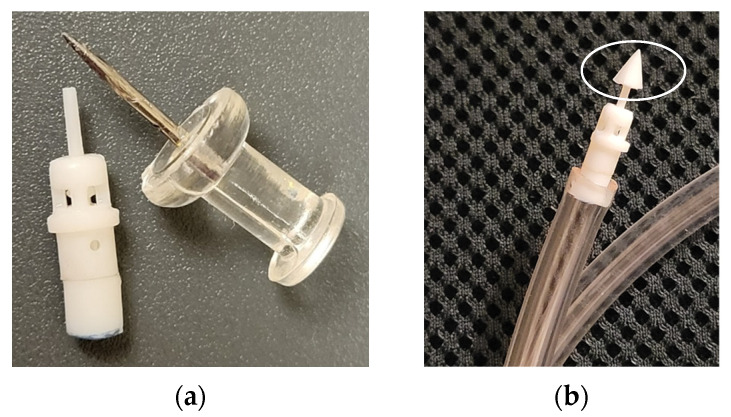
Assembled prototype: (**a**) baseline design (option 1) next to a push pin for scale; (**b**) representative conical cutting tip on the shaft.

The post-processing of printed parts was a multi-step manual process. First, the supports were removed using tweezers and then sanded using sandpaper of grit P150 and smaller depending on the extent of residual stub (aluminum oxide sandpaper, any manufacturer). It was noticed that the printed holes were generally undersized and were enlarged using fine drill bits of nearest size (Augenweide, micro-twist hand drill, Amazon, Seattle, WA, USA) to ensure the tight assembly of the components. If the surfaces were not flat, fine grit sandpaper was used to level the surface to ensure proper bonding using glue (Gorilla superglue, no drip gel) during assembly. Prior to glue application, the rotation of rotor was verified through tactile feedback. If the rotation was not smooth, the mating surfaces were smoothened using the fine grit sandpaper. The alignment of base and cap was also critical for the proper rotation of the output shaft. This was achieved by aligning the outside diameter of the cap and base before the glue dried. An assembled device is shown in Figure 4a. A standard push pin is placed next to the assembled motor for highlighting its relative size. When a cutter is attached to the motor output shaft it rotates, providing a cutting action similar to drilling or reaming. For the first iteration, a conical reamer was printed. Figure 4b shows a representative conical cutting tip attached to the shaft.

**Figure 5:**
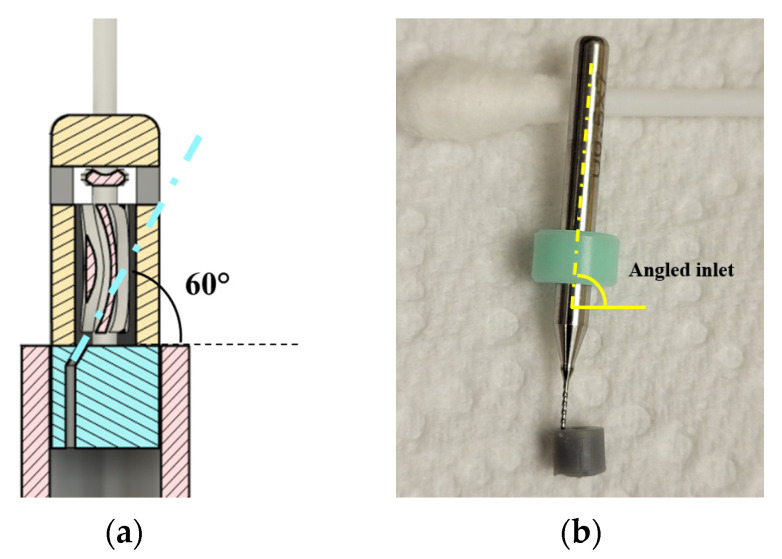
(**a**) Base design with inlet for tangential water entry into the blades; (**b**) base with angled inlet shown using a drill bit inserted into it for visualization.

The design option 2 with tangential inlet was also prototyped and assembled. The inlet diameter was 400 µm angled at approximately 60° to horizontal in the CAD model as shown in Figure 5a. The base with angled inlet is shown in Figure 5b with a drill bit inserted into the inlet for visualization. During SLA printing, the holes were usually undersized, and the inlet hole had to be enlarged using the nearest drill size. The angle of the printed hole was used as a pilot for enlarging it. However, it can be seen in Figure 5b that the angle of the inlet was greater than the nominal value of 60° in the physical prototype.

**Figure 6:**
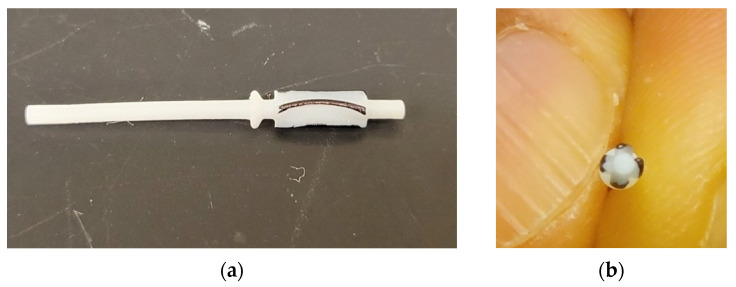
Four-blade rotor (2 mm outer diameter at the blades): (**a**) side view; (**b**) bottom view.

The design option 3 prototypes with 2 inlets look identical to the baseline design after assembly and hence are not shown here. Figure 6 shows the rotor with 4 blades (option 4). The blades are highlighted in black for visualization.

### 2.2. Experimental Setup

For testing the 4 mm prototypes, water was used as working fluid even though the prototypes can be operated using air. This was primarily to utilize the setup developed in a previous work [43]. The building water supply was used in the setup during experimentation. The RPM was measured using a tachometer (model number: LH900RF, EHDIS, Amazon, Seattle, WA, USA). The retroreflective tape was provided along with the tachometer by the manufacturer. The tachometer was mounted on a stand at a distance of 4 to 6 inches from the device. Pressure was monitored throughout the duration of the experiment using a pressure gauge (3847K71, 0 to 200 PSI, 0 to 30 PSI, McMaster-Carr, Elmhurst, IL, USA). For lower flowrates, a 0 to 30 PSI (least count 0.5 PSI) gauge was used, and for higher flowrates, a 0 to 200 PSI (least count 5 PSI) gauge was used.

**Figure 7:**
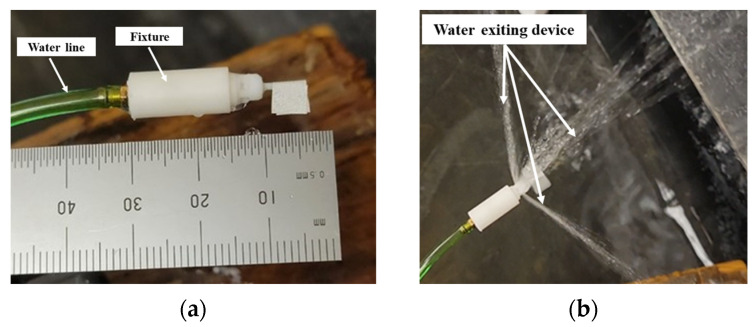
(**a**) Prototype connected to waterline using a fixture; (**b**) water splatter at device outlets.

Because of the small size of the device (4 mm OD), it could not accommodate the inlet barb fitting for water connection. A fixture had to be designed to connect the waterline to the prototype as shown in Figure 7a. A custom vibration damping clamp was used (part number: 3015T999; McMaster-Carr) to secure the device. There was very limited space for attaching retroreflective tape for angular velocity measurements using a handheld tachometer. The flag was sized approximately 5 mm × 5 mm to fit the laser spot. This configuration was tested first, since the RPM was successfully measured using a flag and drum in earlier experiments [43]. However, when the flag was attached to the shaft as shown in Figure 7a, the measurements were not consistent and had many outlier readings. It was suspected that the water exiting the device caused splatter (Figure 7b), which interfered with the tachometer laser.

**Figure 8:**
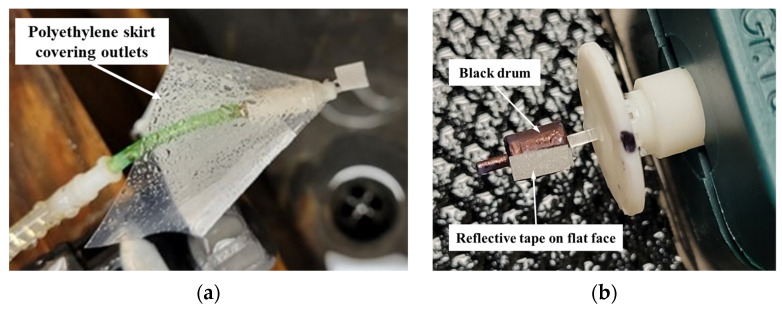
(**a**) Polyethylene skirt to contain splatter at outlets; (**b**) black drum with reflective tape on flat face.

**Figure 9:**
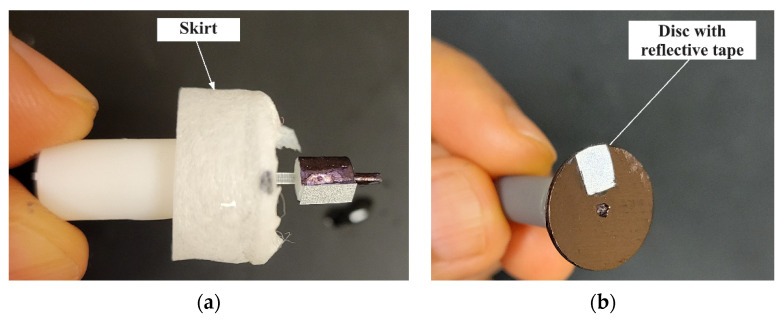
(**a**) Skirt to prevent water splatter from interfering with laser; (**b**) prototype with disc instead of a drum.

The outliers were still present with a polyethylene skirt fitted to the prototype as shown in Figure 8a to overcome the issue of water splatter. It was hypothesized that the flag did not provide good contrast, i.e., dark vs. reflective regions for a strong signal (reflected laser) to be detected by the tachometer. To improve the repeatability of measurement, a flange was incorporated on the cap to divert the waterflow away from the retroreflective tape and the tape was attached to a flat face on the black cylinder, referred to as a “drum” (4 mm in diameter and 5 mm in height). This configuration is shown in Figure 8b. This increased the signal to noise ratio for the tachometer. The strategy was effective at lower flowrates but required an additional skirt for measurements at higher flowrates, as shown in Figure 9a.

**Figure 10:**
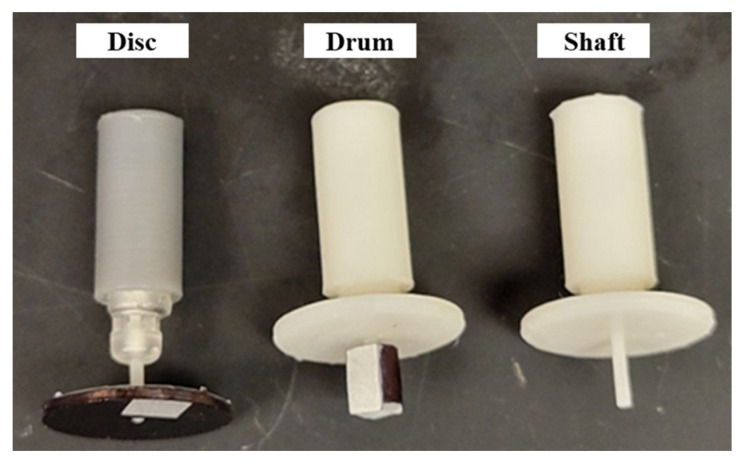
Disc, drum, and plain shaft arrangements to measure RPM.

**Figure 11:**
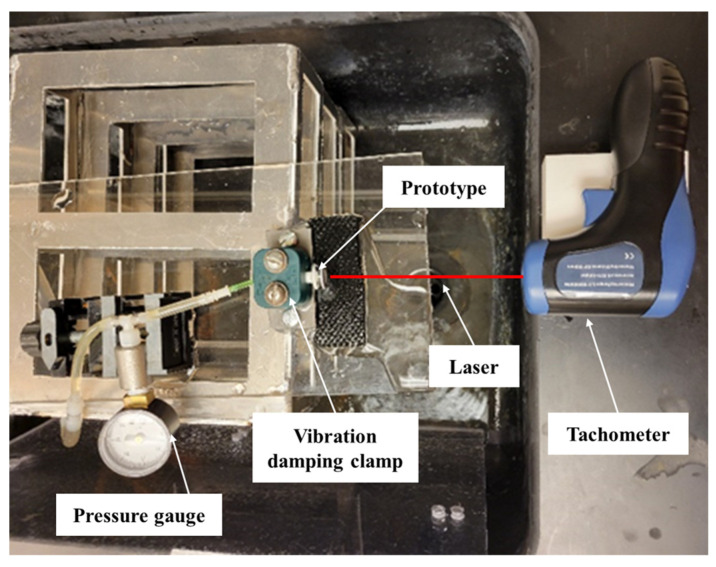
Setup for measuring RPM using “Disc”.

In some prototypes, a disc was used for RPM measurements (Figure 9b and Figure 10). This experiment design was inspired by the setup of Furukawa, Y., et al. [29], where a gear was used to intermittently reflect a laser. The number of times a reflective signal is detected depends on the number of gear teeth and the rotor speed. In our case, the disc (15 mm diameter, 1 mm thickness) was colored black and a single piece of reflective tape approximately 5 mm × 3 mm was glued along the radius. When using the disc, the tachometer was positioned on the marble block (Figure 11) and was kept steady.

**Figure 12:**
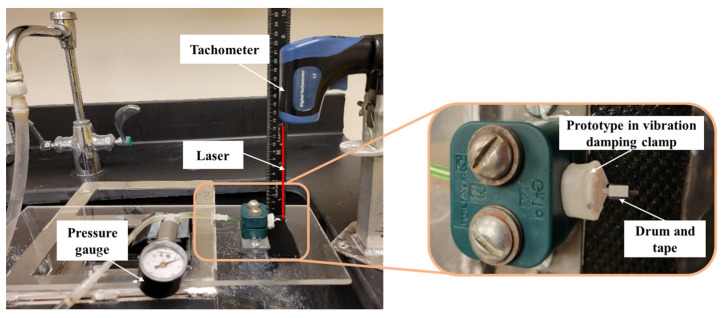
Setup for RPM measurement using “Drum”. The prototype was held in position by a vibration-damping clamp for angular velocity measurement.

The setup for RPM measurement using drum is shown in Figure 12. The laser was continuously ON using the “Auto” function, and the “Record” feature was used to sample motor RPMs via the tachometer at a rate of one reading per second.

### 2.3. Computational Fluid Dynamics (CFD) Model

The CFD models of the baseline geometry, i.e., design option 1, were created in ANSYS CFX version 2022 R2. These models were three-dimensional with turbulence and incorporated fluid structure interaction. The k-ϵ turbulence model in conjunction with rigid body solver was used to obtain a solution. Immersed solid methodology was used to model the interaction between rotor and fluid. The rotor was constrained except for rotation about Z-axis and the angular velocity was an output of the simulation for a specified inlet flowrate. The authors have successfully used this modeling methodology with a prior prototype [43]. Total simulation time was 0.05 s with a timestep of 5 µs. The initial fluid velocity was set to 0 m/s at a relative pressure of 0 PSI. The simulation was run until a steady-state angular velocity of rotor was observed.

The mesh study was performed using an inlet water flowrate of 0.01 kg/s and outlet was defined as an opening at atmospheric pressure. An inlet flowrate of 0.0036 kg/s was also used in the simulations. Experiments were conducted at these exact flowrates for comparison. To estimate the approximate internal resistance torque in the device, a special case was run where the rotor RPM was defined as an input and the rotor torque was measured as the output. For this special run, an inlet flowrate of 0.003 kg/s was defined, and the outlet boundary condition was same as above. The internal resistive torque was estimated by comparison with the experimental data.

## 3. Results and Discussion

In this section, the following results are presented: (Section 3.1) Experimental results obtained from benchtop testing of the prototypes; (Section 3.2) Mathematical equations to calculate peak torque for both perpendicular and tangential inlets (design options 1 and 2); and (Section 3.3) Numerical simulations results.

### 3.1. Experimental Results

Different prototypes were tested either using the drum or the disc arrangement but not simultaneously on the same prototype. It must be noted that the drum or disc once glued in place cannot be interchanged without breaking the shaft. Device-to-device fluctuation can be attributed to differences in surface roughness as the components were manually processed for support removal after printing. These differences will be reduced in the next iteration when micro-additive manufacturing is used. Since the building water supply was used in the test setup, non-uniformity in flowrate was observed when the pressure was set at a particular value. This was due to random pressure variations smaller than the resolution of the instrument (the least count of pressure gauge). If it was observed that the pressure drifted from the set value during the test, it was adjusted back to the desired pressure by opening or closing the faucet valve. This manifested into fluctuations of prototype RPM and was quantified during data analysis as the spread (standard deviation) in recorded data points. The summary of the experimental data for all prototypes is presented in Table 1. The baseline design prototype, with inlet perpendicular to the blades and a rotor with five blades, was extensively tested on the benchtop at various flowrates and the corresponding rotor angular velocities were recorded. The drum arrangement was used for RPM measurement. The experimental data for this prototype were used for developing the numerical model using ANSYS CFX, the results of which are presented in Section 3.3. Data were collected with and without ambient light, and no significant differences in the RPM data were observed. The average angular velocity when the experiment was repeated with ambient light was 10,259.7 RPM, and when it was conducted in a dark room, the average rotor angular velocity was 10,213.7 RPM at 7 PSI pressure and a flowrate of 3.7 mL/s.

Consistent rotor output was achieved using prototypes of design options 2, 3, and 4 as well. The disc arrangement was used for recording the RPM data. For design option 2 (tangential inlet, rotor with five blades), a total of 32 data points were collected in four sets of eight measurements each. For the prototype with two inlets perpendicular to the blades (option 3), a total of 30 data points were collected in five sets of six measurements each. With the prototype that has single inlet and a rotor with four blades (option 4), a total of 31 data points were collected in three sets of eight measurements each and one set of seven measurements.

Table 1 summarizes the descriptive statistics of the experimental data collected for all the design options. Supplementary Spreadsheet S1 presents the raw data collected during the experiments.

The rotor RPM is sensitive to the internal resistance of the device, i.e., the higher the internal friction, lower the RPM. Due to the imperfections during post-processing of printed parts and manual device assembly, a device-to-device variation in friction is expected. Despite the differences in friction, the experimentally measured rotor angular velocities for design options 2, 3, and 4 were significantly lower compared to option 1 for a given inlet flowrate. Theoretically, option 3 with dual inlets would produce higher torque at lower RPM as the kinetic energy from two water jets is transferred to the rotor. Since torque was not experimentally measured, this hypothesis can be verified through numerical simulations in future. The experiments demonstrated a reliable shaft output for all four design options, and option 1 was selected for further miniaturization.

### 3.2. Mathematical Equations to Calculate Peak Torque

#### 3.2.1. Inlet Perpendicular to the Rotor Blades (Design Option 1)

This section describes the mathematical model developed for calculating the peak torque by treating the problem as an interaction between a water jet and curved plate. The curved plate has a linear velocity U due to the rotor rotation. If the angular velocity (ω) of rotor is known, U can be easily calculated using radius (r) × ω. The following assumptions were made:

(a) The blades are smooth with negligible friction.
(b) The jet spreads symmetrically after impingement and maintains constant contact with the blade surface.
(c) The jet interacts with one blade at a time and is perpendicular to it.

**Figure 13:**
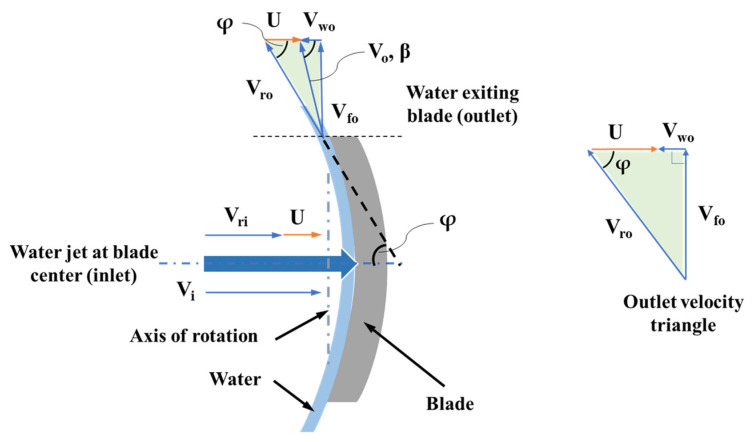
Velocity components at inlet and outlet of the blade.

In Figure 13, the velocities of the incoming jet are denoted by a subscript “i” and the outlet velocities are denoted by subscript “o”. The force acting on the blade can be calculated by the change in momentum of the incoming water jet and the jet exiting the blade.

The relative velocity at inlet (V_ri_) can be expressed in terms of blade linear velocity (U) and inlet jet velocity (V_i_), as shown below in Equation (1):

V_ri_ = V_i_ − U (1)

At the blade outlet, the relative velocity of jet is V_ro_ and the absolute velocity of jet exiting the blade is V_o_. The whirl and flow components of the absolute velocity are denoted by V_wo_ and V_fo_. The angle made by the absolute velocity V_o_ with horizontal is β. If the blade angle is φ, the jet is deflected by an angle (180 − φ) degrees. Considering the velocity triangle at the blade outlet (Figure 13), Equation (2) is obtained:

V_wo_ = V_ro_ Cos φ − U (2)

The force due to momentum being transferred from jet to the blade is given by Equation (3):

(3) $$F=\dot{m}\;(V_{\mathrm{ri}}+V_{\mathrm{ro}}\mathrm{Cos}\varphi )$$

where $\dot{m}$ is the effective jet mass flow interacting with the blade. Substituting Equations (1) and (2) into (3), we obtain:

(4) $$F=\dot{m}\;(V_{i}+V_{\mathrm{wo}}),\;\mathrm{for}\beta <90{}^{\circ}$$

It must be noted that relative velocity at inlet (V_ri_) is equal to relative velocity at outlet (V_ro_) since the blades were assumed to be frictionless. As the rotor radius (r) is known, using the force value from Equation (4), the peak torque can be computed. Equation (5) is for computing the force when β > 90°. This case occurs when the rotor RPM is high near the no-load condition. The effective mass flowrate can be calculated using Equation (6). Based on the geometry, the effective mass flowrate was calculated by multiplying the total mass flow by a factor ϵ, i.e., the proportion of the jet that is perpendicular to the blade. This factor ϵ was calculated to be 0.7 using the prototype geometry. Water density is denoted by ρ, and the jet cross-section area is represented by variable A.

(5) $$F=\dot{m}\;(V_{i}-V_{\mathrm{wo}}),\;\mathrm{for}\beta >90{}^{\circ}$$

(6) $$\dot{m}=\rho \epsilon AV_{\mathrm{ri}}$$

**Figure 14:**
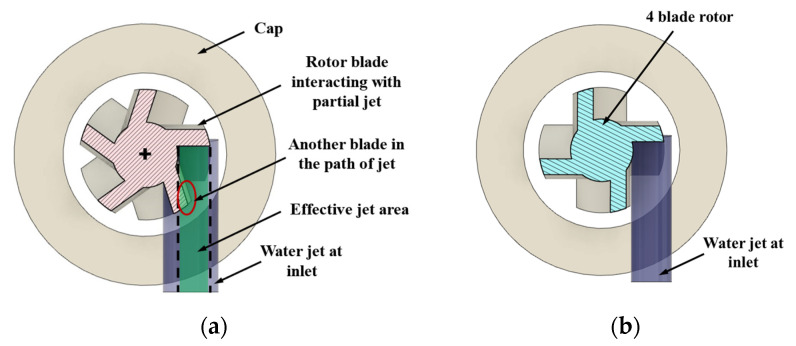
Water jet interaction with blades: (**a**) five-blade rotor; (**b**) four-blade rotor.

In the model, there were five blades on the rotor. Due to the relative position of inlet and the blades, the jet interacts with two blades simultaneously in the instantaneous position for peak torque. However, it is perpendicular to one blade and the tip of the second blade is in the jet’s path, as shown in Figure 14a. For the rotor with four blades (Figure 14b), the entire jet can be perpendicular to only one blade at the instantaneous position that produces peak torque. However, when the rotor is rotating, the next blade cuts into the jet’s path. For future designs, the relative position of inlet jet w.r.t blade, the inlet diameter and number of blades can be adjusted for optimizing the torque.

Peak torque (T) on the rotor can therefore be calculated by using Equation (7), where F is the force due to jet and r is rotor radius:

T = F r(7)

For a flowrate of 0.0036 kg/s measured experimentally, peak torque values were calculated using the equations and compared with the simulation results in Section 3.3. An inlet diameter of 850 µm, an effective rotor radius of 740 µm, and a blade angle of 61.6° were used for calculations. The calculations were in good agreement with the simulation results.

#### 3.2.2. Inlet Tangential to the Rotor Blades (Design Option 2)

**Figure 15:**
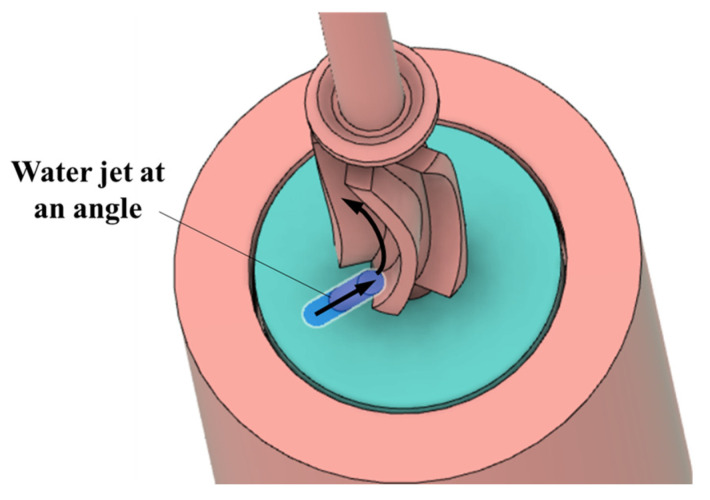
Water jet at an angle to the blade.

A similar mathematical model was also developed for the case where the inlet jet was tangential to the blade (Figure 1b). Figure 15 shows a CAD image of the angled inlet jet.

**Figure 16:**
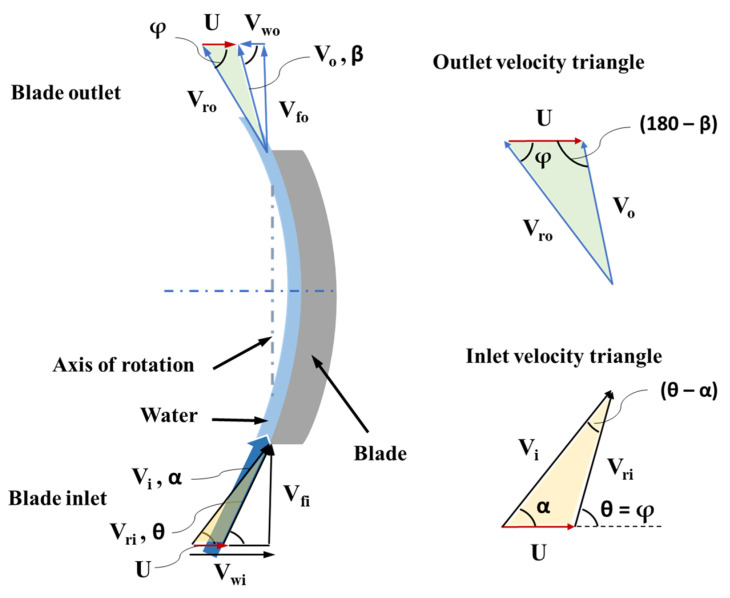
Velocity triangles for the inlet jet tangential to the blade.

Figure 16 shows the velocity triangles at the blade inlet and outlet. The same notation as above was used for the velocities. Since the blade is symmetric, blade angle at inlet θ is equal to the blade angle φ at the outlet.

The jet inlet angle (α) is an important design parameter, and it can be calculated using the velocity triangles at inlet (Equation (8)). The known parameters are the blade angles (θ = φ), jet deflection angle (180 − φ), flowrate through the device, and the RPM of the rotor. V_ri_ is equal to V_ro_ since the blades were assumed to be frictionless. Blade linear velocity can be calculated as rotor radius times the angular velocity (U = rω). The jet velocity (V_i_) can be calculated using the inlet diameter and the mass flow through the device. By applying the sine rule at the inlet velocity triangle, Equation (8) is obtained:

(8) $$\frac{V_{i}}{\sin\left(180-\varphi \right)}=\frac{U}{\sin\left(\theta -\alpha \right)}$$

At the outlet velocity triangle, applying the sine rule gives Equation (9):

(9) $$\frac{V_{o}}{\sin\varphi }=\frac{V_{ro}}{\sin\left(180-\beta \right)}$$

Also, V_wo_ can be calculated using Equation (10):

(10) Vwo=Vo cos⁡β

The angle created by the absolute velocity of the jet at the outlet with the horizontal, β, can be calculated using Equations (9) and (10). And for β at less than 90 degrees, the force on the blade is given by Equation (11):

(11) $$F=\dot{m}\;(V_{wi}+V_{wo}),\;\mathrm{for}\beta <90{}^{\circ}$$

Since the entire jet interacts with the blade, the mass flowrate ($\dot{m}$) can be calculated using ϵ equal to 1 in Equation (6). Using this force, the torque can be estimated by multiplying with the rotor radius (Equation (7)).

### 3.3. Numerical Simulations Results

The results of ANSYS CFX 3D transient simulations of the baseline design (option 1—a single inlet and a rotor with five blades) are presented in this section. In a mesh independence study, the grid was varied from 0.9 million to 3.3 million elements. In each case, a torque of 50 µNm (initial estimate) was applied to the rotor in the mesh study. This represents the realistic scenario of non-zero internal resistance on the rotor. The relative error in rotor steady-state angular velocity between a mesh with 2.6 million elements and the finest mesh with 3.3 million elements was 1.5%. Hence, a mesh with 2.6 million elements was chosen as optimal for subsequent analyses. The change in absolute value of average rotor steady state angular velocity between these two grids was 120.2 rad/s (1148 RPM). It can be noted that in Table 1, the spread (range) in RPM from the baseline prototype experiments was 2568 for a flowrate of 10 mL/s (0.01 kg/s) at 40 PSI, which was higher than the modeling uncertainty of 1148 RPM.

From the analysis with rotor RPM as simulation input (4500 RPM), the average torque was determined to be 6.6 µNm. Therefore, for rest of the analyses, the resistance torque on the rotor, whether frictional resistance or external resistance, in the range of 0 to 10 µNm was used for a mass flow of around 0.003 kg/s (3 mL/s) at the inlet. The applied torque in the simulation where the RPM output matches the experimental RPM is the internal resistance of the device.

**Figure 17:**
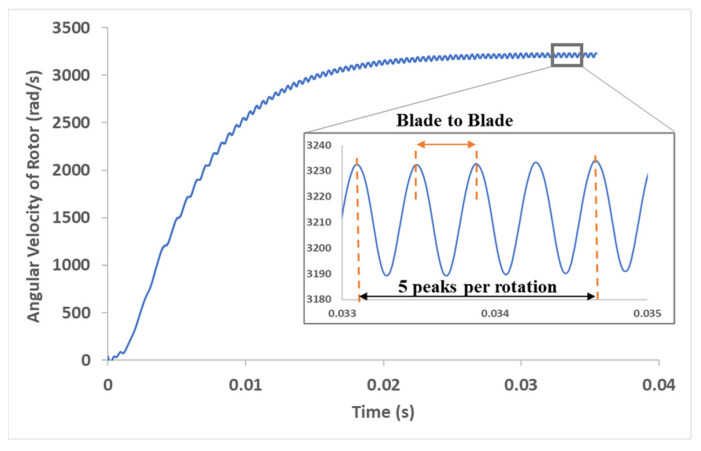
Rotor angular velocity (rad/s) vs. time (s). Inset shows periodicity in rotor angular velocity relative to blade orientation w.r.t inlet jet. The angular velocity peaks when the jet is perpendicular to the blade.

The peak torque is achieved when the orientation of the blade is perpendicular to the inlet water jet. As the blade rotates, the angle at which the jet strikes the blade changes and the torque decreases. Similarly, there is an inherent periodicity in RPM depending on the blade orientation w.r.t inlet as shown in Figure 17. When the blade is perpendicular to the jet, a peak in RPM is observed. Since the design had five blades on the rotor, five peaks were observed per rotation (360 degrees) of the shaft. The frequency of RPM peaks depends on the time required for one shaft rotation. In Figure 17, a torque of 5 µNm was applied to the rotor with an inlet flowrate of 0.0036 kg/s. The frequency of RPM peaks in this case was calculated to be 2558 Hz. The amplitude of the waveform was 40 rad/s (382 RPM). This was much smaller compared to the spread in the experimental measurements (1418.2 RPM, Table 1). The tachometer sampling frequency was 1 Hz. Therefore, with the current instrumentation, it is not possible to resolve and measure the periodicity observed in the simulations. This illustrates the usefulness of the CFX model in characterizing the internal flow and the output shaft response.

The average rotor angular velocity for a flowrate of 3.6 mL/s was 10,604.5 ± 159.1 RPM from the simulation for a torque of 9 µNm on the rotor. The corresponding experimentally measured value was 10,593.4 ± 778.8 RPM for the same flowrate.

The error bars on predicted RPM values from the analysis were calculated based on the 1.5% discretization error as described previously. The standard deviation calculated from the experimental data accounts for random variability and measurement error (Table 1). Assuming the experimental data are normally distributed, ±3 standard deviations (3 × 259.6 = ±778.8 RPM) would account for 99.7% of the data. Qualitatively and quantitatively, the model is in good agreement with experimental results to within experimental uncertainty. Therefore, for the prototype that was tested, and the model predicted an internal resistive torque of 9 µNm on the rotor since no other external load was applied to the shaft during experimentation.

**Figure 18:**
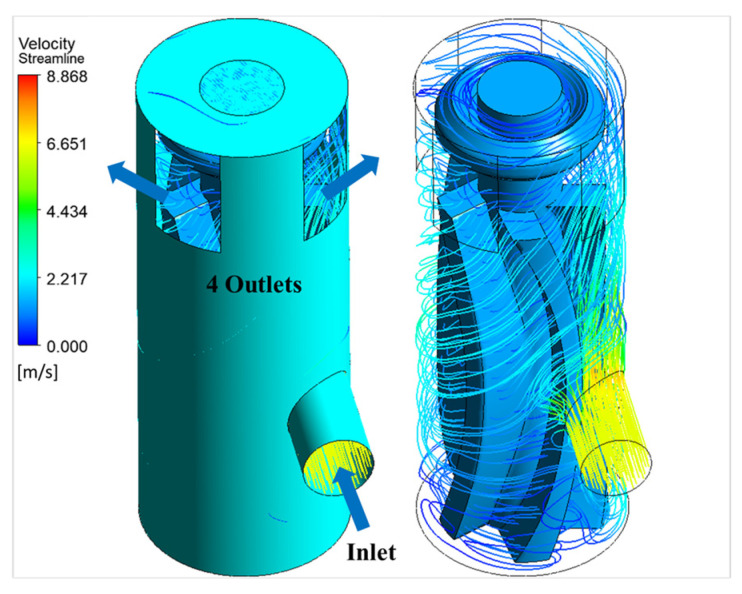
Three-dimensional velocity streamlines from the simulation with and without the outer wall.

**Figure 19:**
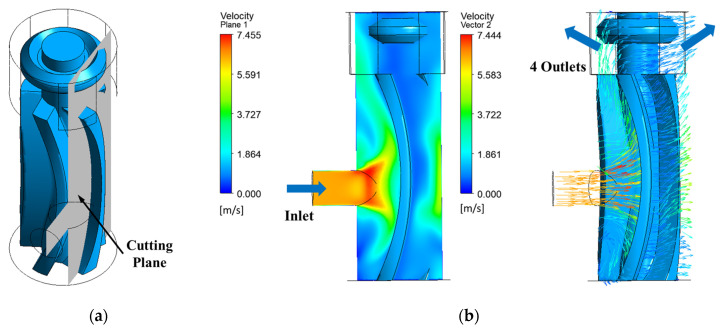
Flow at the blade: (**a**) plane through inlet; (**b**) velocity contour and vectors on the plane through inlet cutting across the blade at an intermediate timestep.

Figure 18 shows the velocity streamlines from the simulation with a resistive torque of 9 µNm on the rotor. Figure 19 shows the velocity contours and vectors along a plane passing through the center of inlet. Intuitively, the jet is expected to be split evenly into two streams and spread along the blade surface, imparting its momentum to the blade. This movement of fluid along the blade curvature can be visualized in Figure 19b. Since the fluid exits from the top through the four openings and since there are no exits at the bottom in this design, the portion of jet which is directed towards the base impinges on the bottom face (base). It is then recirculated by the rotating blades before exiting the device. The fluid does not exit evenly through the openings either, there are slight differences in mass flow through individual openings due to the circulation. Understanding the internal flow helps in optimizing the outlet placement in future designs. For instance, placing additional outlets at the bottom can be explored for reducing the recirculation inside the device. Supplementary Video S1 shows the animation of the flow through the device and the corresponding development of rotor movement.

**Figure 20:**
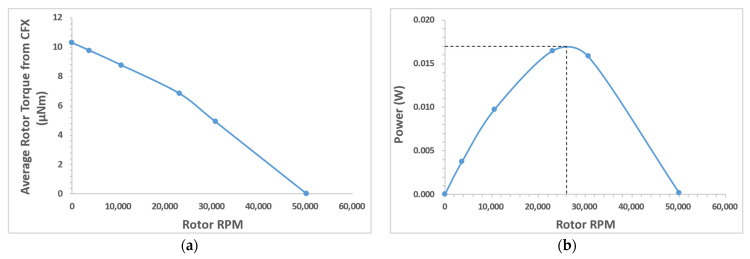
Performance curves generated from ANSYS CFX simulations: (**a**) torque vs. RPM; (**b**) power vs. RPM. The discrete datapoints are connected by smooth lines only to serve as a visual aid.

Performance curves were generated for the miniature motor using the CFX models. Figure 20a shows torque (µNm) vs. angular velocity (RPM). The stall torque was predicted to be 10.3 µNm. The zero-torque case is an ideal case where there is no internal resistance, i.e., friction is zero, and there is no load on the device. Figure 20b shows the power (W) vs. rotor angular velocity (RPM). The peak power output from the cutting module was found to be 17 mW at 26,000 RPM.

Table 2 shows the comparison of peak torque from the mathematical model (Section 3.2.1) and from the CFX computational model. In the CFX model, the rotor had five blades. Therefore, for one complete rotation there would be five torque peaks. An average of at least 10 torque peaks (two rotor rotations) from the CFX model was considered when comparing with the peak torque from the mathematical model. The equations tend to overestimate the torque due to the underlying assumption that the jet is interacting with one blade at a time. However, the second blade obstructs a portion of the jet, reducing the “effective” jet area, as shown in Figure 14. This factor would impact the torque calculated using the equations. Even with this inherent error, the mathematical model was in good agreement with the computational model. The CFX model considers the effect of turbulence in the fluid domain for calculating the torque and predicts the RPM and torque fluctuations in addition to the peak torque, whereas the mathematical model calculates the instantaneous maximum torque value.

In general, a sampling frequency of every 20 timesteps was used for collecting the torque data in the simulations. However, at higher RPMs (near-zero resistance torque), the sampling rate must be increased to capture the “true” torque peaks and avoid aliasing.

Even though numerical modeling has been limited to the baseline design for the current discussion, for the target 2 mm design, the design options 2, 3, and 4 will be analyzed for a potential improvement in torque or RPM output.

### 3.4. Comparison with Dental Burr and Other Miniature Motors

Dental handpiece manufacturers such as NSK Dental [44], KaVo Dental [45], and W&H [46] produce various high speed air turbines with a power greater than 20 W for dental procedures. One such air turbine handpiece from NSK dental, S-Max pico [47], was chosen for computing the torque due to its small size. The head’s outside diameter is 8.6 mm, and therefore, the rotor is smaller than 8.6 mm. It has a rated power of 9 W and a rotational speed range of 380,000 to 450,000 RPM with an average angular speed of 415,000 RPM (43,458.7 rad/s). The torque can be estimated as 0.21 mNm using the manufacturer’s provided data of power and RPM.

The rotor size in the 7 to 8 mm range is of particular interest for direct comparison with a prior design from a previous publication [43]. The torque (at a peak power of 0.058 W, 440 rad/s) was estimated to be 0.132 mNm (compared to 0.21 mNm for the dental handpiece) for that prototype. It must be noted that the prototype was not optimized for performance and was manufactured using SLA printing process with minimal post-processing of parts. Whereas the dental turbine is a sophisticated precision instrument for optimal output. Despite the rudimentary design of miniature hydraulic turbine prototype, the estimated torque was comparable. The difference in power was primarily due to the high rotational speed of dental turbine, which is supported by specialty bearings on both ends of the turbine shaft.

As the rotor was scaled down from 8 mm to 2 mm, the estimated peak torque decreased from 0.132 mNm (at a peak power of 0.058 W) to 6.24 µNm (at a peak power of 0.017 W). It is crucial to note that the power did not decrease at the same order of magnitude as the torque since the rotational speed of smaller device was higher and it compensated for the decrease in torque to some extent. Also, the flowrate was higher through the 8 mm rotor prototype than through the 2 mm rotor prototype.

Table 3 compares the different motor types in terms of overall dimensions, torque, RPM, etc., based on the values reported in the literature. The RPM of the prototype hydraulic motor presented in this paper was much higher than those of the ultrasonic and ECF motors and is comparable to that of electric motors.

## 4. Conclusions

A miniature hydraulic motor with a ~4 mm outside diameter was successfully designed and manufactured using additive manufacturing. This is a crucial intermediate design step in an ongoing miniaturization effort to develop a 2 mm OD hydraulic motor for biomedical applications. The device reported here establishes the feasibility of achieving reliable mechanical work output from miniature hydraulic motors. Multiple design variants were prototyped with different inlet and blade configurations, i.e., (1) baseline design with single inlet perpendicular to the blades (5×); (2) single inlet tangential to the blades (5×); (3) dual inlets with a 5-blade rotor; (4) single perpendicular inlet with a 4-blade rotor. The prototype devices were tested to show consistent rotational output. A benchtop experimental setup was developed, and a procedure was established for testing the prototypes. RPM and flow data were obtained and reported for prototypes of all the four design versions. Even though all the design options provided a reliable output, the baseline design with single inlet perpendicular to the blades was chosen for the preliminary prototyping of 2 mm OD device. The baseline design exhibited higher shaft RPM for a given flowrate. However, it must be noted that the prototypes were assembled manually and there is device-to-device variation in the internal resistance.

A numerical model was developed using ANYSYS CFX and was used to predict the performance characteristics of the prototyped 4 mm OD miniature hydraulic motor with a single inlet perpendicular to the blades (baseline design). The peak power output from the prototype motor was found to be 17 mW at 26,000 RPM. The stall torque was predicted to be 10.3 µNm. The numerical model was developed and validated by the RPM data from the experiments. This model can be used for estimating the internal resistance (flow friction) of the device. The average rotor angular velocity for a flowrate of 3.6 mL/s was 10,604.5 ± 159.1 RPM from the simulation for a torque of 9 µNm on the rotor. The experimentally measured average RPM value was 10,593.4 ± 778.8 at the same flowrate.

Mathematical models were also developed for the designs with inlet perpendicular and tangential to the blades. The peak torque values calculated using the mathematical equations were compared to the values from the numerical simulations for the baseline design and were found to be in good agreement.

Future work involves manufacturing and testing to-scale 2 mm OD device prototypes. Preliminary to-scale prototypes have been fabricated using micro-additive manufacturing, the specifics of which are beyond the scope of this paper. Repeatable assembly and experimentation are foreseeable challenges due to the small size of the device.

In summary, we present a 2:1 scale prototype of the cutting module and establish the proof of concept for consistent mechanical work from a biocompatible miniature hydraulic motor with output characteristics comparable to motors currently available in a similar size range. Lessons learned from the current 4 mm motor development will enable miniaturization by halving the device diameter to 2 mm and further exploring the possibility of scaling down again. This critical milestone brings the target 2 mm OD design a step closer to fruition.

## 5. Patents

Invention disclosure is being processed though the University of Cincinnati Office of Innovation (reference number: UC 2023–121, June 2023).

## Figures and Tables

**Table 1:** Descriptive statistics for the experimental data.

Design Option	Pressure (PSI)	Average Flowrate (mL/s)	Sample Size	Mean RPM	St. Dev. *	Minimum RPM	Maximum RPM	Range
1 (single perpendicular inlet, five blades)	6.5	3.6	52	10,593.4	259.6	9944.8	11,363.0	1418.2
	11.5	5.6	25	17,346.6	287.2	16,666.0	17,821.0	1155.0
	24.5	8.4	5	29,724.4	273.2	29,411.0	30,101.0	690.0
	26	8.7	24	32,675.3	653.2	30,822.0	33,333.0	2511.0
	40	10.1	27	42,596.6	625.9	40,910.0	43,478.0	2568.0
2 (single tangential inlet, five blades)	11	2.6	32	576.4	11.4	556.8	604.2	47.4
3 (dual perpendicular inlets, five blades)	6.5	3.7	30	5005.1	46.1	4909.9	5070.5	160.6
4 (single perpendicular inlet, four blades)	11.5	2.1	31	4122.6	13.4	4100.7	4145.7	45.0

* Standard deviation (RPM).

**Table 2:** Torque comparison: CFX vs. equations for the baseline design.

Rotor RPM	Average Peak Torque of the CFX Model (µNm)	Peak Torque of the Math Model (µNm)
50,106.7	2.23	2.56
30,654.6	6.51	6.56
23,003.4	8.29	8.65
10,604.5	9.74	12.6
3683.9	10.4	15.2

**Table 3:** Comparison of different motor types.

Motor Type	Diameter (mm)	Torque (µNm)	RPM	Power (mW)
Ultrasonic [32]	4.5 (OD)	19.6	2000	4.1
Pelonis Electric [25]	4 (OD)	7.8	15,000	12.3
ECF [35]	5 (ID)	16.3	892	0.4
Proposed Hydraulic	4 (OD) ^+^	6.24 *	26,000 *	17 *

^+^ At the tip; * peak power, refer to Figure 20.

## Data Availability

The data presented in this study are available on request from the corresponding author. All the data are not publicly available due to the invention disclosure being processed.

## Supplementary Materials

The following supporting information can be downloaded at: https://www.mdpi.com/article/10.3390/mi15010131/s1. Spreadsheet S1: Experimental Data, Video S1: Video of rotor movement w.r.t flow.
